# Supplementary material for: Spike-in enhanced phosphoproteomics uncovers synergistic signaling responses to MEK inhibition in colon cancer cells
Source: Nat Commun. 2025 May 27;16:4884. doi: 10.1038/s41467-025-59404-y (PMC12106795; doi:10.1038/s41467-025-59404-y)
Supplement: Supplementary file 1 — Supplementary Information [file 41467_2025_59404_MOESM1_ESM.docx]

### Supplementary Information to “Spike-in enhanced phosphoproteomics uncovers synergistic signaling responses to MEK inhibition in colon cancer cells”

*
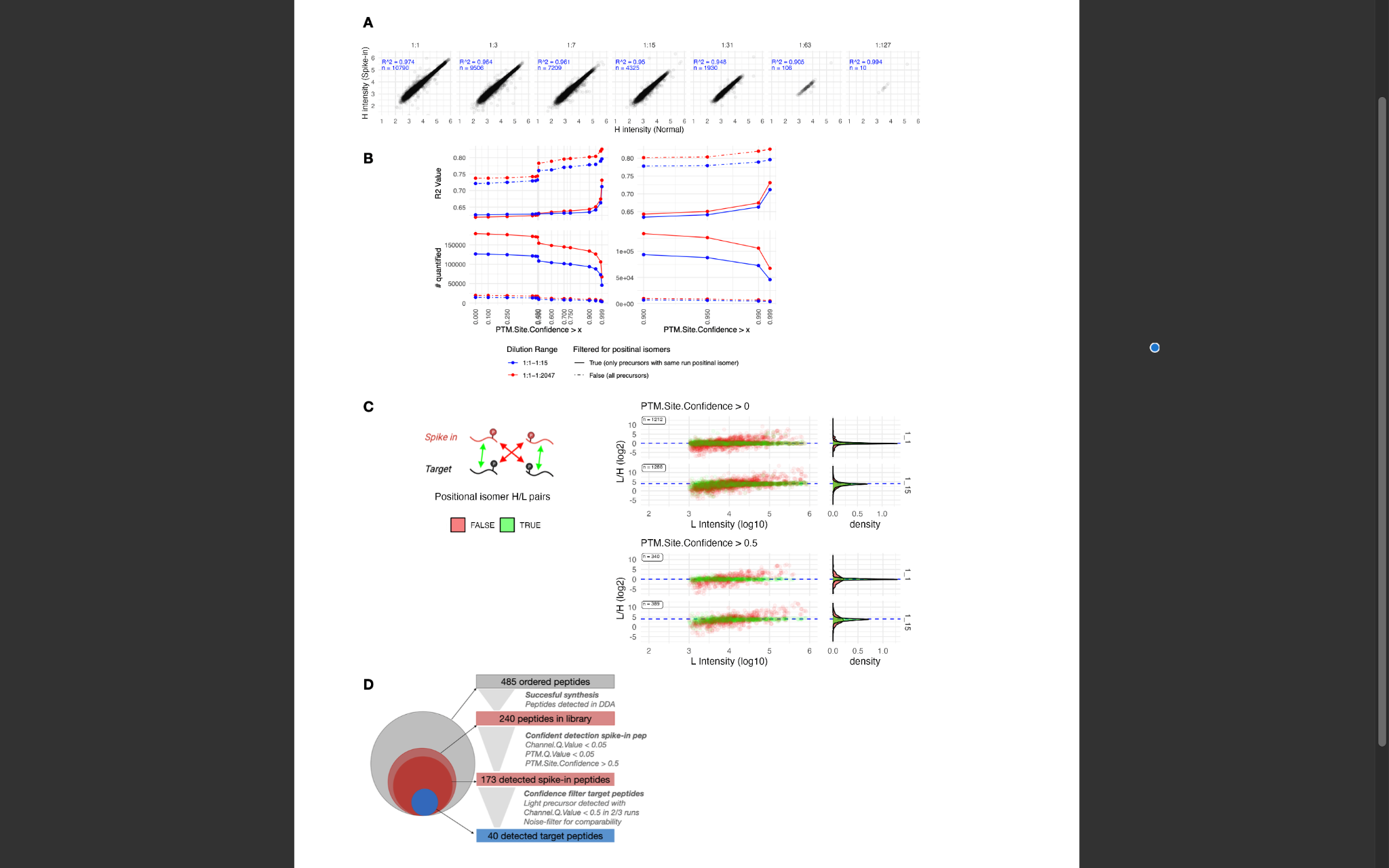
*

**Supplementary Figure 1: Analysis of Quantification Across Method, Dilutions and Filters.** A. Correlation of H (target) intensities between Normal SILAC-DIA and SPIED-DIA, with R² and precursor counts (n) indicated for each dilution factor. B. Effect of PTM.Site.Confidence filtering on R² values for the correlation between expected and observed SILAC ratios, contrasting first four dilutions (1:1-1:15, red) with the full range (1:1-1:2047, blue). Solid lines represent all precursors, and dotted lines represent data filtered for peptides with same run/charge positional isomers. Lower panels reflect the number of precursors that survive filtering. Right panels provide a focused view on the 0.9 to 1. C. Comparison of H/L ratios across channel / same vs different phosphorylation site within positional isomer pairs in 1:1 and 1:15 dilutions (Figure 1C). Data was filtered for peptides with same run positional isomers to visualise how well the measured H/L ratios reflect the expected ratio within a dilution. Ratios are plotted against intensity of reference channel. Channel.Q.Value was kept consistent and PTM.Site.Confidence was varied according to the panel title. D. Illustration of how the 40 target peptides were derived from the initially selected peptides, filtered for confidence in detection and reproducibility across replicates.


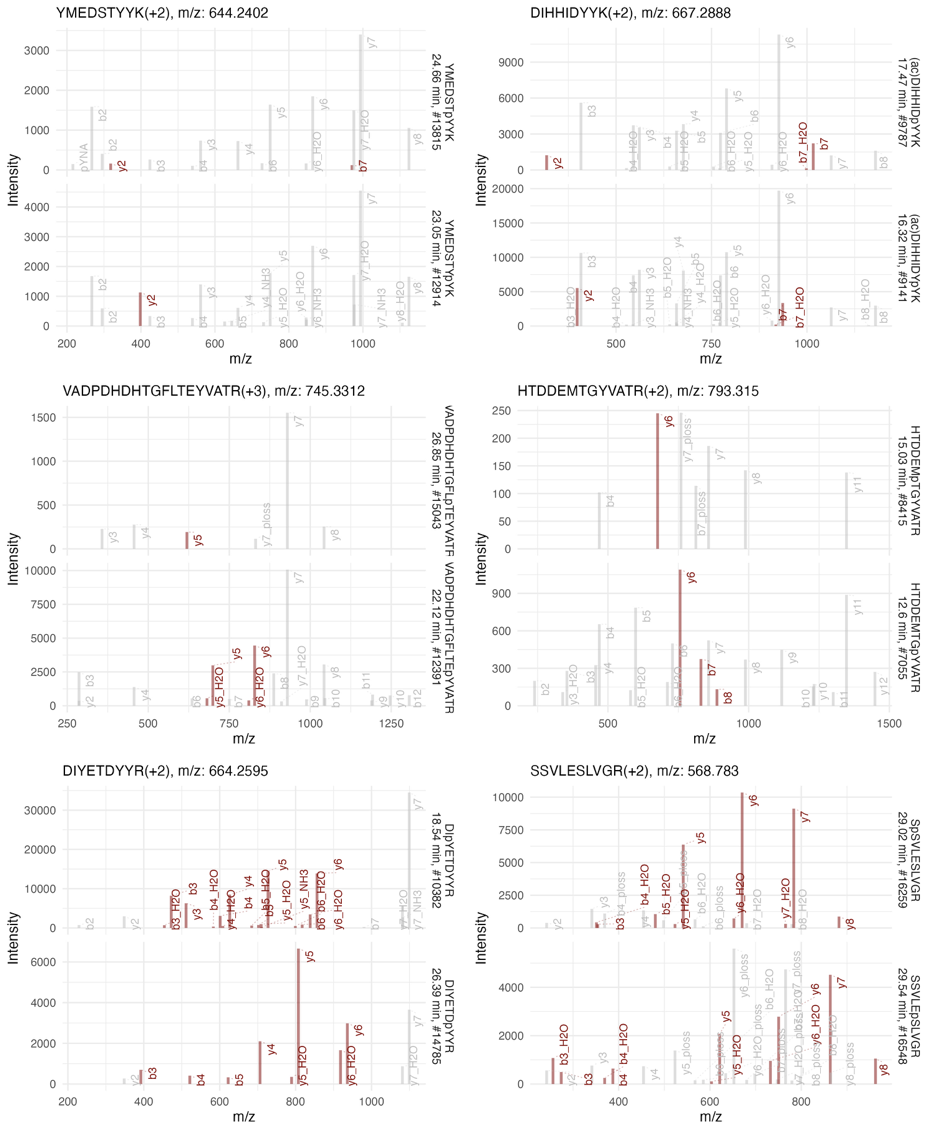


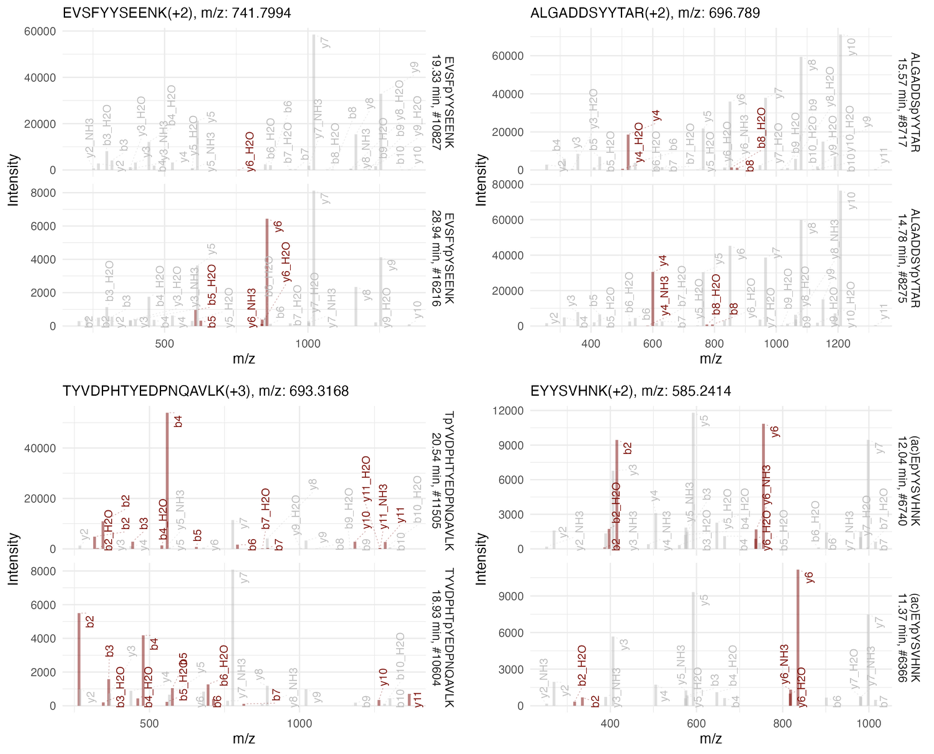


**Supplementary Figure 2: Validation of positional isomeric phosphopeptides in raw data of example sample HCT116 DMSO/GFmix.** Figure represents spectra of selected phosphopeptides. We randomly selected phosphopeptides in heavy form with matching amino acid sequence and other modifications (such as N-terminal acetylation) except for phosphorylation, which is localized at different residues. Peptides are identified in identical charge states. Unmodified sequence, charge status and m/z are represented above plots. Modified sequence, retention time and Bruker raw file spectrum ID are depicted at the left of plots. Fragments are labeled in accordance with fragment m/z in the library. Fragments that are crucial for distinguishing between isoforms are highlighted in red.


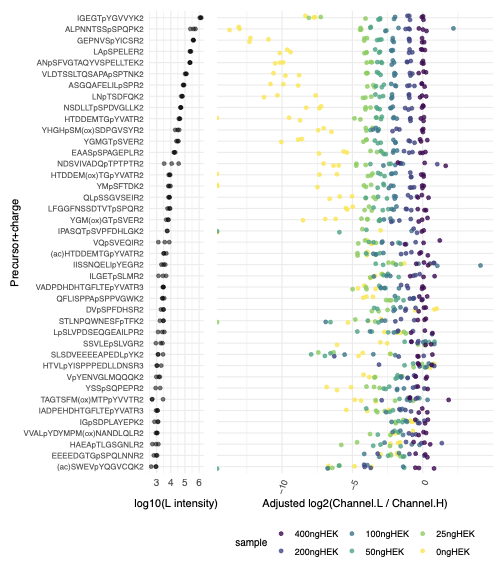
**Supplementary Figure 3: ​​Dilution series quantification analysis in DIA-NN.** Left panel shows intensity in the 400ng condition. Right panel depicts log2 L/H ratio (relative to 400ng condition) per precursor. Precursors are sorted according to intensity in 400ng dilution. Displayed precursors pass the standard SPIED-DIA filtering in the 400ng condition, and additionally need to have a mean light intensity > 10^3 in the 400ng samples.

**
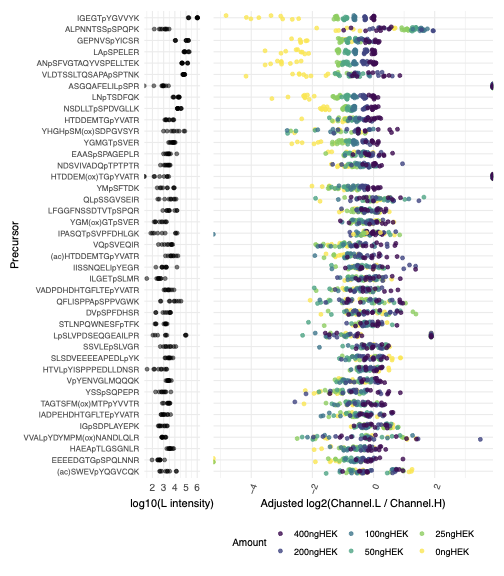
Supplementary Figure 4: Dilution series quantification analysis Skyline**. Left panel shows intensity in the 400ng condition (summed intensity top 3 fragments). Right panel depicts log2 L/H ratio (relative to 400ng condition) per precursor-fragment (top 3 fragments). Precursors are sorted according to order in Supplementary Figure 3.


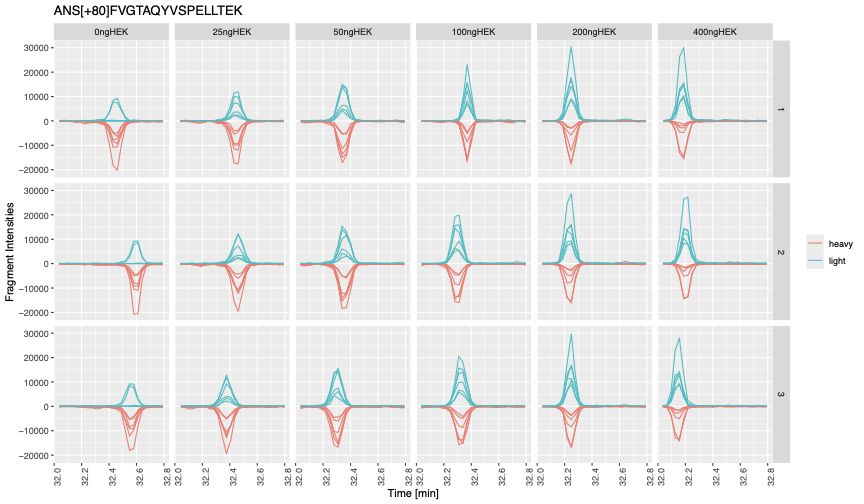


**Supplementary Figure 5: Chromatographic traces for peptide ANpSFVGTAQYVSPELLTEK**. Fragment traces exported from Skyline. Traces of heavy (spike-in) peptide depicted in the red, endogenous in blue. Results for the three replicates (rows) across the dilution series (columns) are shown.


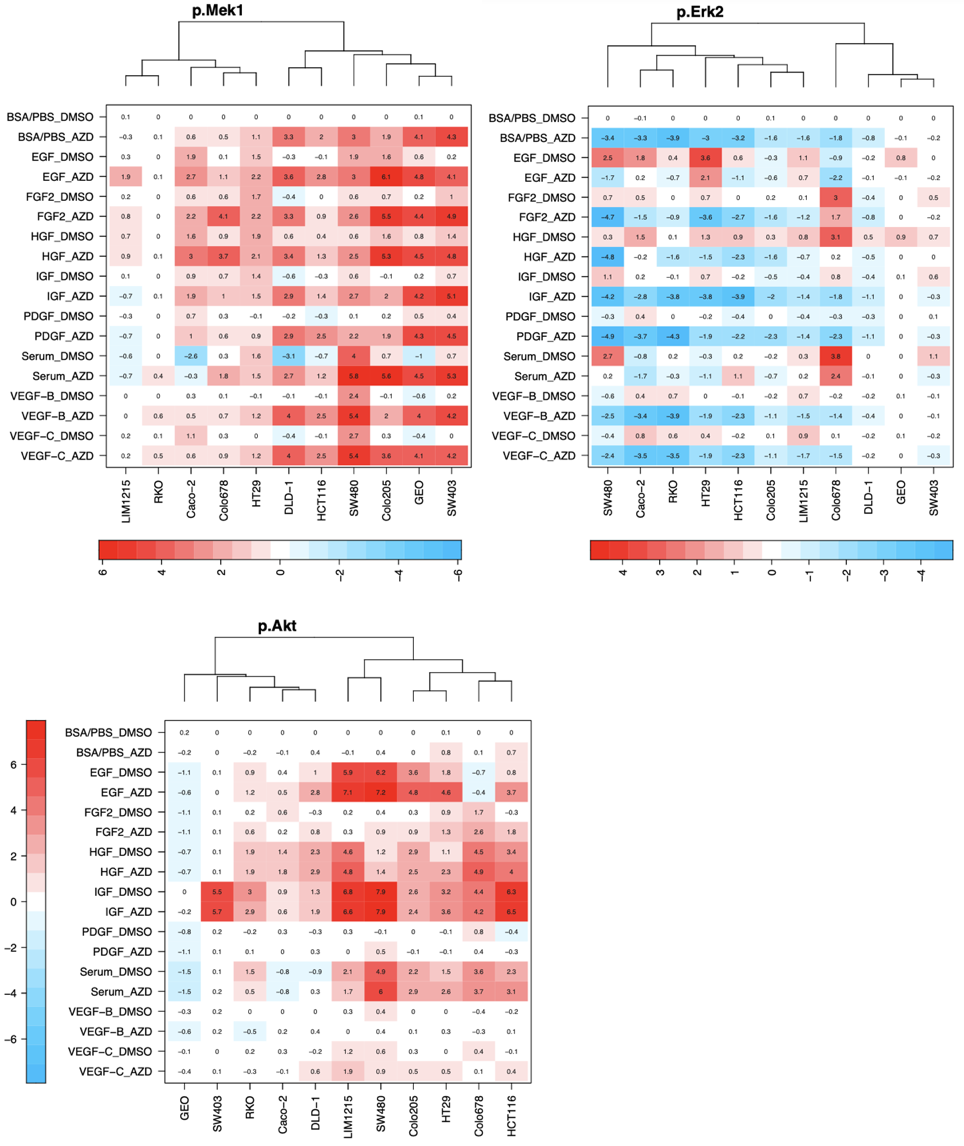


**Supplementary Figure 6: Detailed heatmaps representing luminex results from pAKT, pMEK and pERK2.** Results are separated based on MEKi (“_AZD”) vs control (“_DMSO”) and show intensity normalized to the BSA/PBS_DMSO condition. The expected pattern for pMEK and pERK can be observed. The cell lines per heatmap are hierarchically clustered based on response.
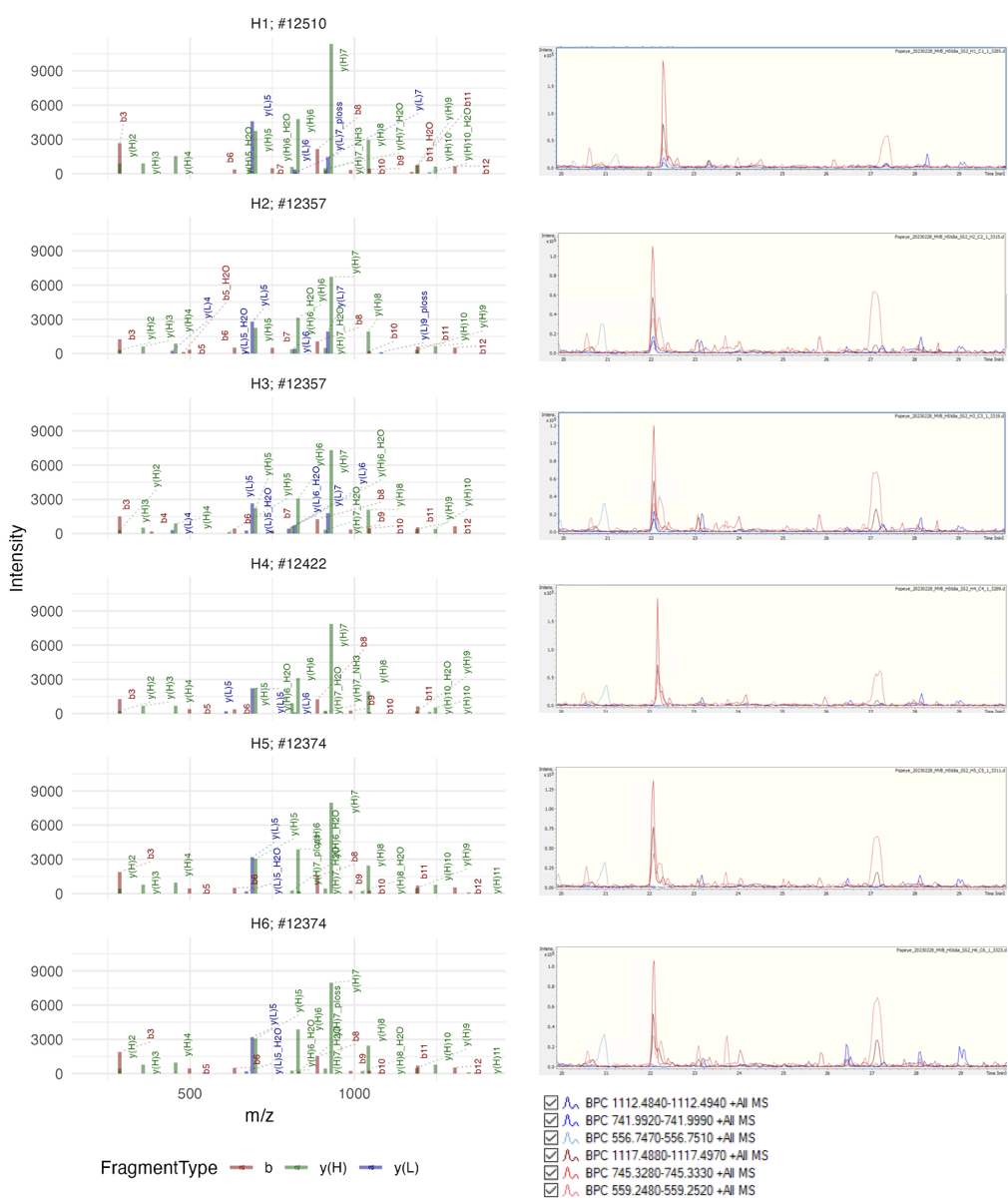

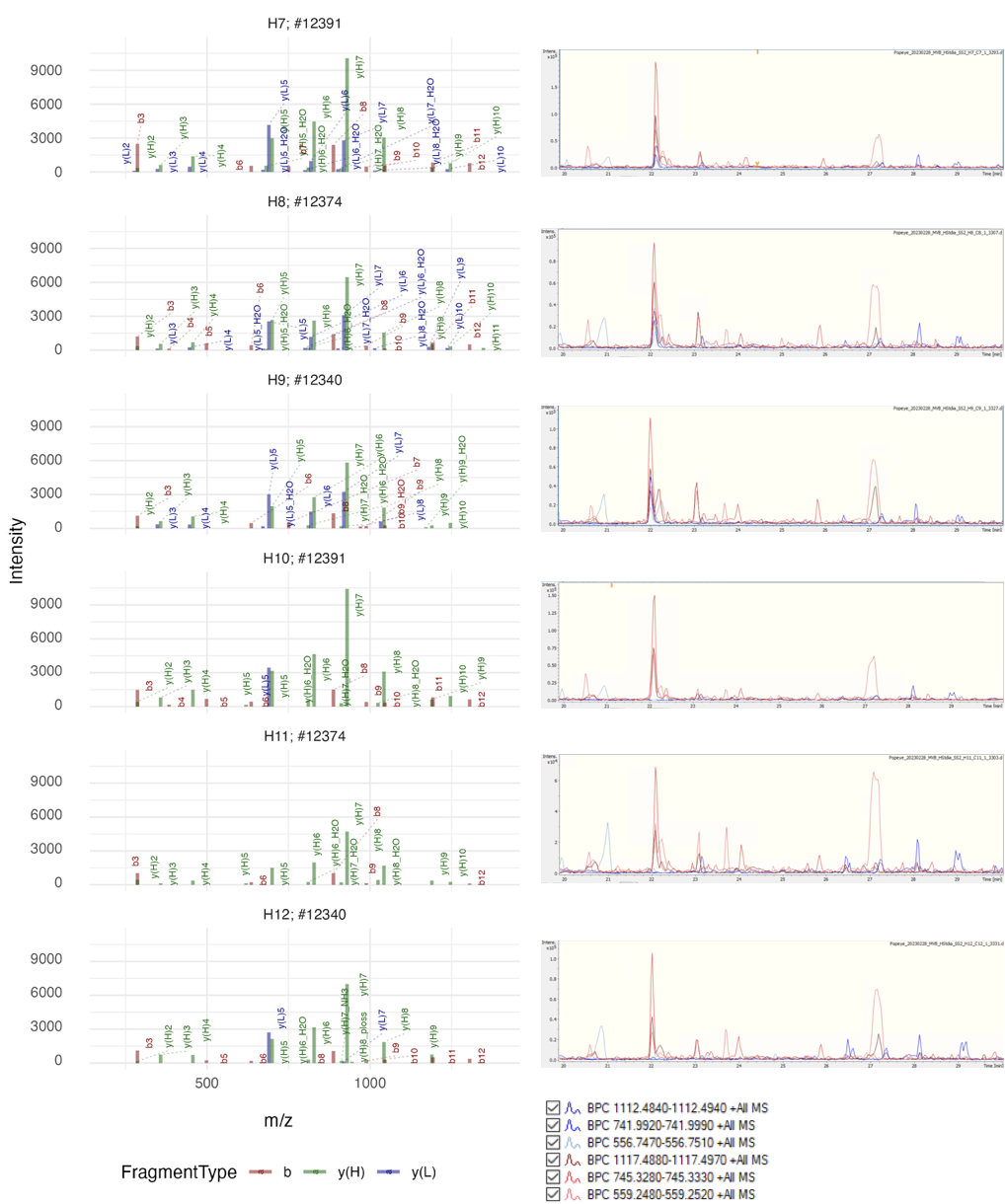


**Supplementary Figure 7: Validation SILAC quantification ERK2 Tyr 187 in MS/MS spectra and MS1 traces in chromatography.** Chromatography, spectrum and SILAC validation VADPDHDHDHTGFLTEpYVATR (3+) in HCT116 (experiment cell-line panel) scan number derived from DIA-NN report.tsv. Only matched ions are shown. Right hand panels show screenshot of traces of MS1 precursor masses. The blue traces depict the endogenous precursor masses (in 2+, 3+ and 4+) and red traces depict heavy or spike in precursor masses.


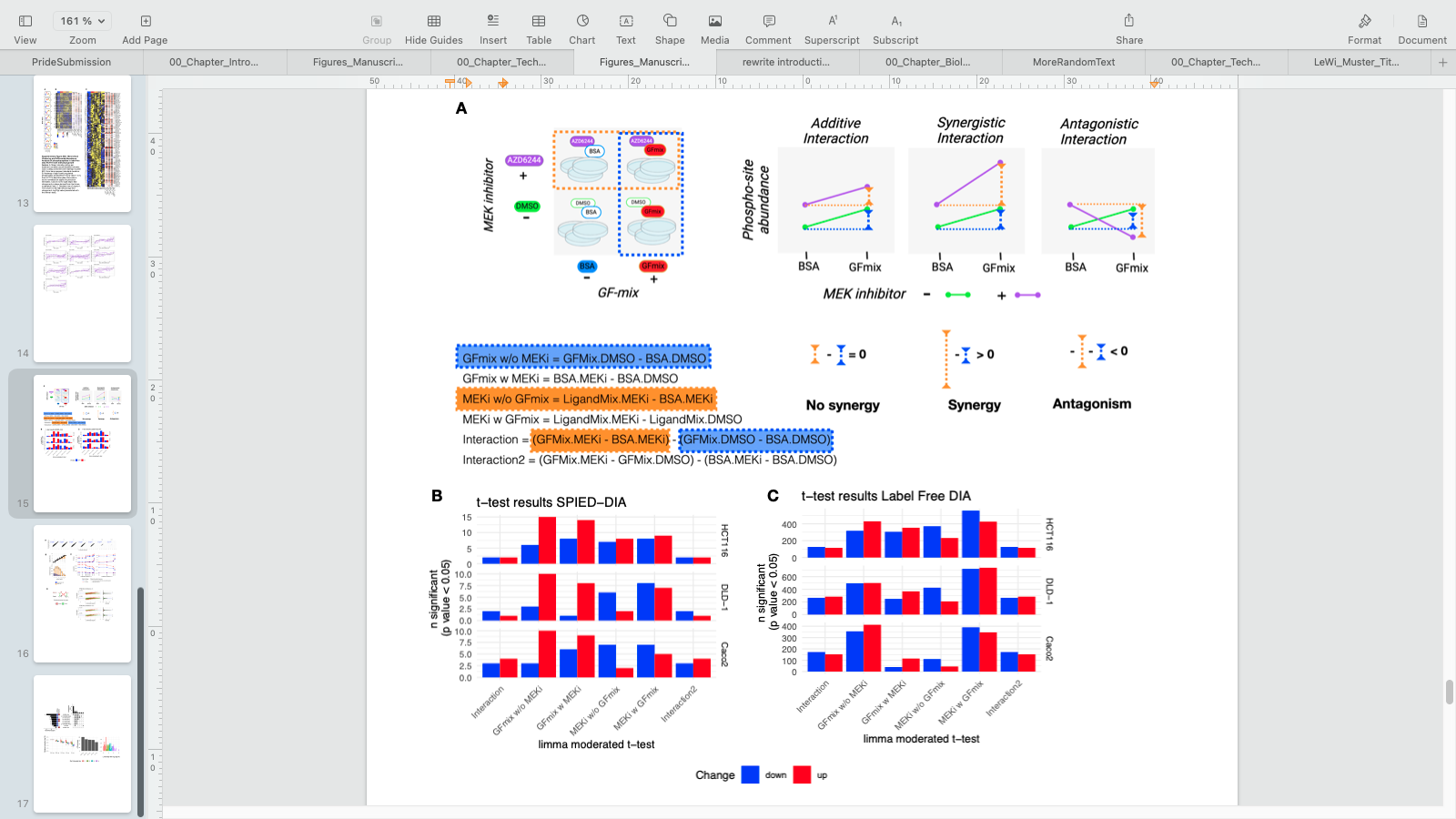


**Supplementary Figure 8. Overview moderated t-test limma to test for synergistic interaction.** A. As described in Materials and methods: Within the factorial design in limma (function “makeContrasts”), contrasts were strategically defined to investigate synergistic effects: the differential impact of the growth factor mix with and without MEKi ("GFmix w MEKi" and "GFmix w/o MEKi"), and conversely, the effect of MEKi with and without the growth factor mix ("MEKi w GFmix" and "MEKi w/o GFmix"). Potential synergistic interactions were explored through an "Interaction" contrast. “Interaction2” was included in analysis as a control but not shown in manuscript. A linear model was fitted to the data and Bayesian statistics (“ebayes” function) were then applied to estimate variance among the precursors, employing moderated t-statistics, resulting in a logFC and (adjusted) p value per test. A positive logFC in the interaction term indicates synergistic interaction, and a negative logFC indicates antagonistic interaction. B. Results moderated t-test as defined in limma in the analysis of the SPIED-DIA data (on precursor level). C. same as B, for the label-free data.


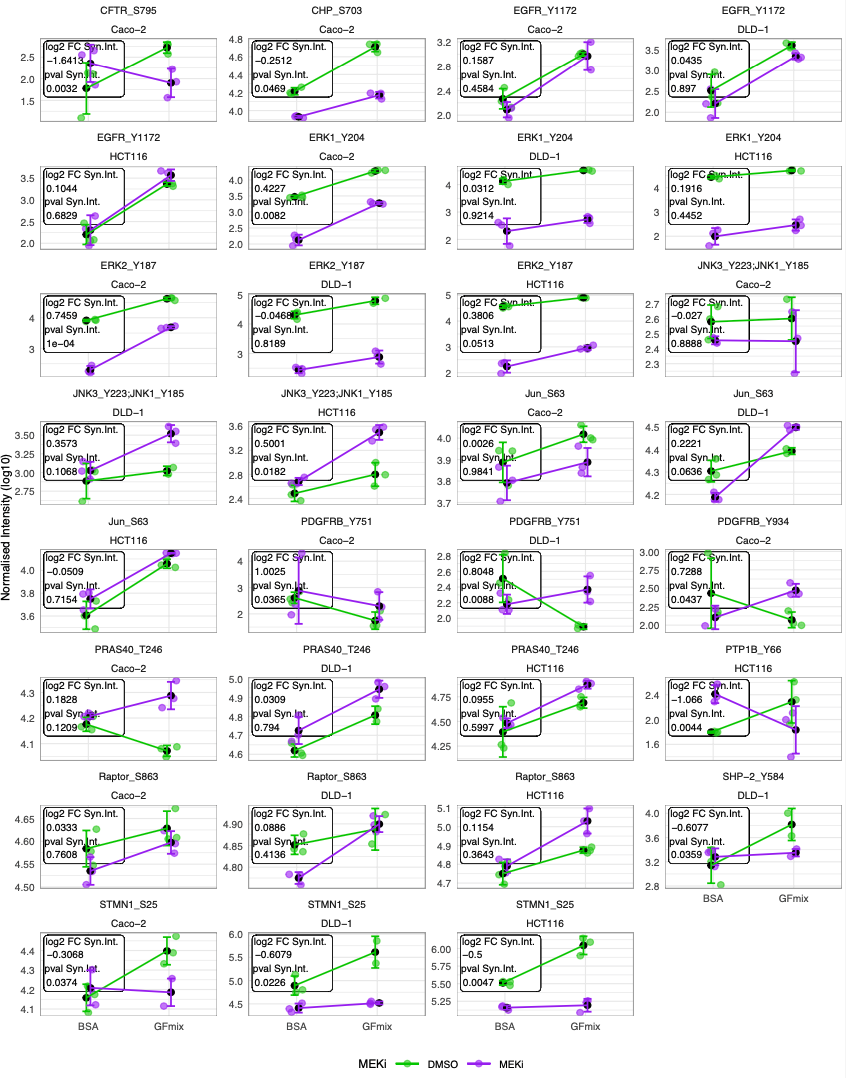


**Supplementary Figure 9. Detailed overview intensity as derived from SPIED-DIA of the phosphosites mentioned in the manuscript**. Per phosphopeptide the precursor with the lowest p-value was selected. Only precursors with at least 2 out 3 identification per condition were included in this plot.


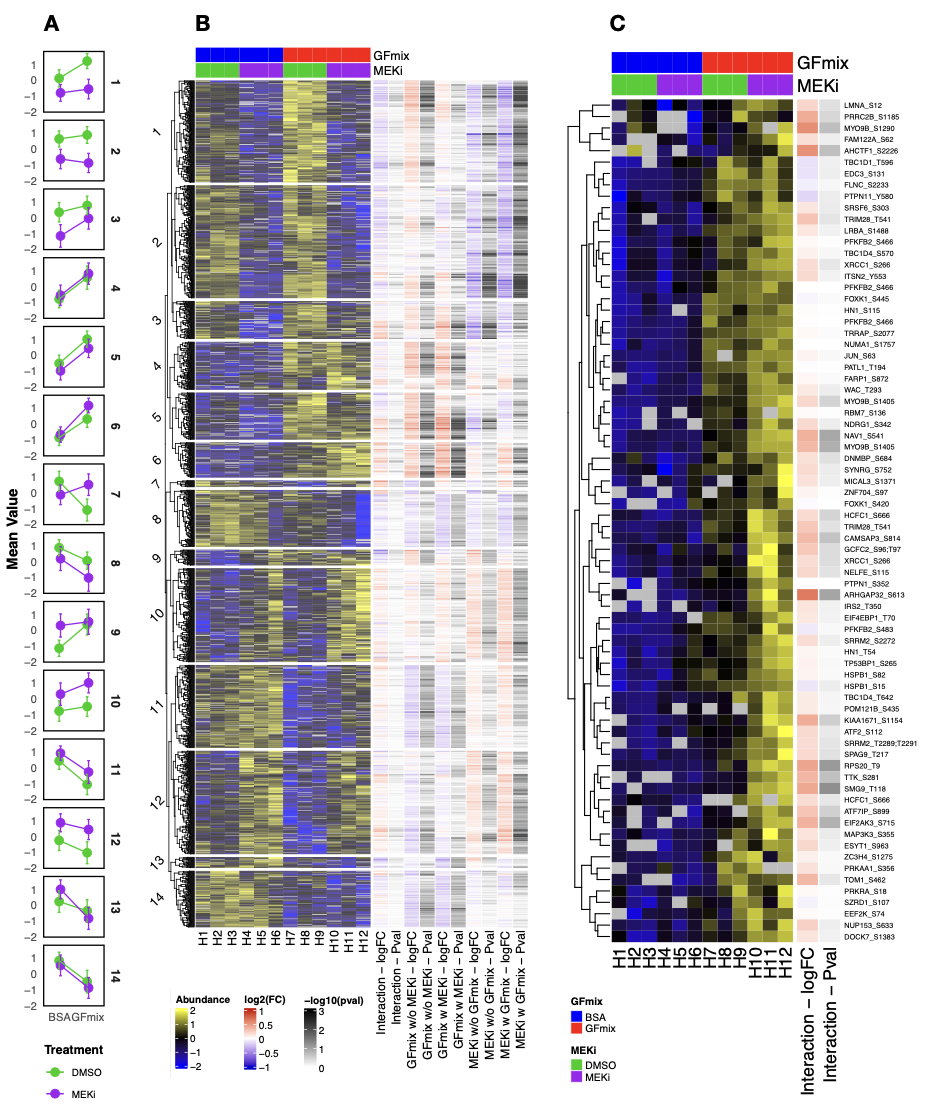


**Supplementary Figure 10: Hierarchical Clustering and Differential Abundance Analysis of phosphopeptides in label-free data HCT116** A. Mean intensity profiles per treatment condition across identified clusters. Color coding consistent with heatmap in panel B. Error bars represent standard deviation. B. Heatmap of significantly regulated phosphosites (moderated F test p-value < 0.1) from HCT116 label-free data. Row-wise z-score normalization applied to precursor intensities. Column to the right depict log2 fold change and BH corrected p-values derived from the limma moderated t-test. C. Detailed view of cluster 6. The column to the right shows log2 fold change and -log10(adj. p-value) associated with the interaction term.


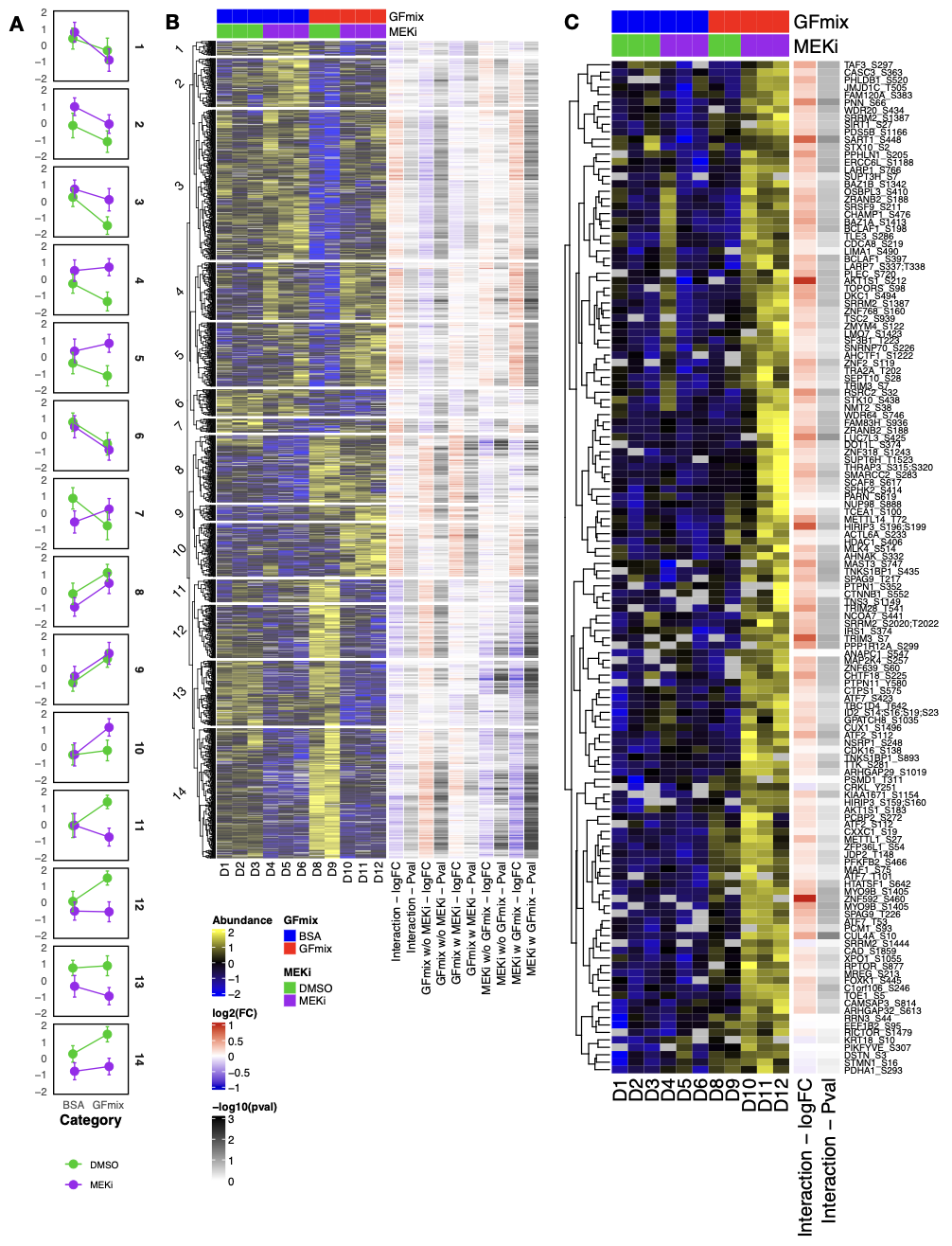
**Supplementary Figure 11: Hierarchical Clustering and Differential Abundance Analysis of phosphopeptides in label-free data DLD-1** A. Mean intensity profiles per treatment condition across identified clusters. Color coding consistent with heatmap in panel B. Error bars represent standard deviation. B. Heatmap of significantly regulated phosphosites (moderated F test p-value < 0.1) from DLD-1 label-free data. Row-wise z-score normalization applied to precursor intensities. Column to the right depict log2 fold change and BH corrected p-values derived from the limma moderated t-test. C. Detailed view of cluster 10. The columns to the right show log2 fold change and -log10(adj. p-value) associated with the interaction term.


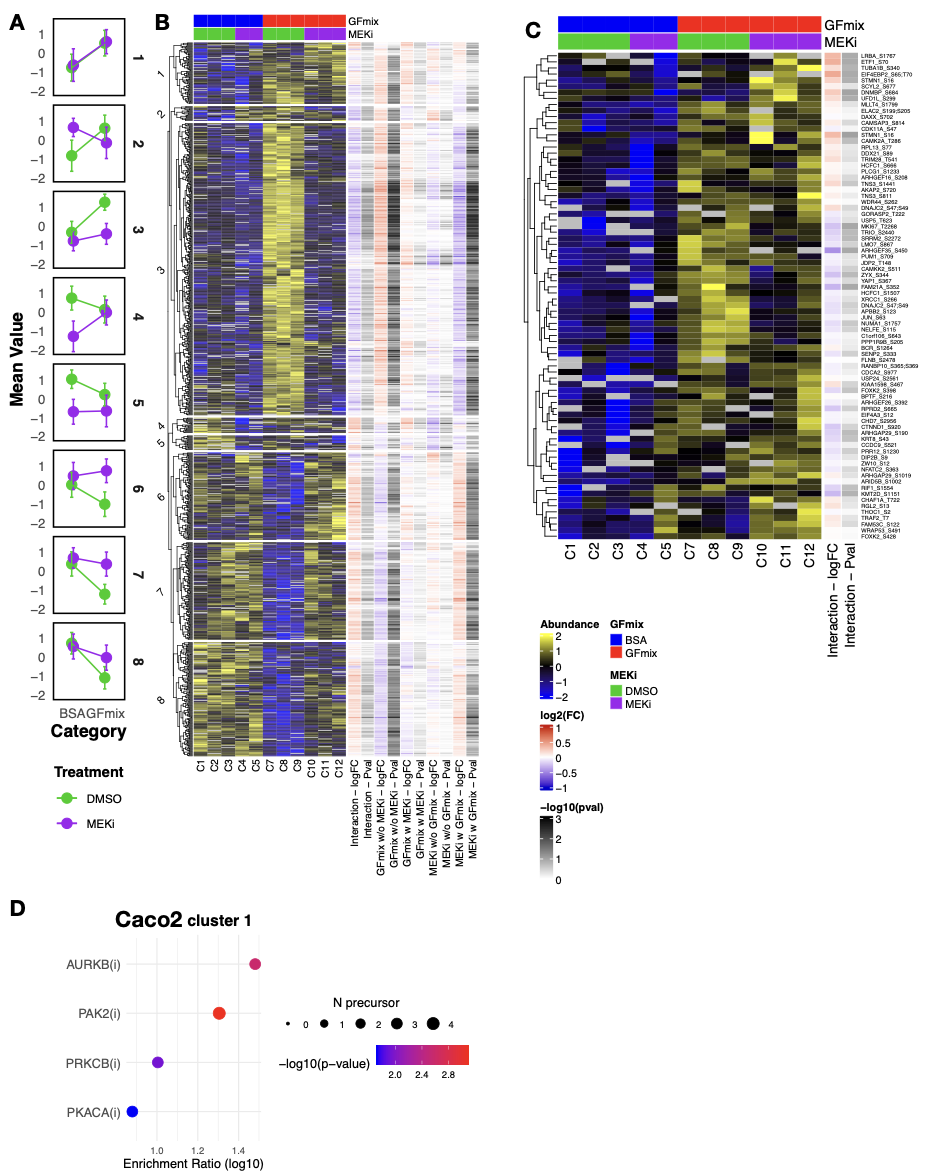


**Supplementary Figure 12: Hierarchical Clustering and Differential Abundance Analysis of phosphopeptides in label-free data Caco2** A. Mean intensity profiles per treatment condition across identified clusters. Color coding consistent with heatmap in panel B. Error bars represent standard deviation. B. Heatmap of significantly regulated phosphosites (moderated F test p-value < 0.1) from Caco2 label-free data. Row-wise z-score normalization applied to precursor intensities. Column to the right depict log2 fold change and p-values (BH corrected) derived from the limma moderated t-test. C. Detailed view of cluster 1. The columns to the right show log2 fold change and -log10(adj.p-value) associated with the interaction term.


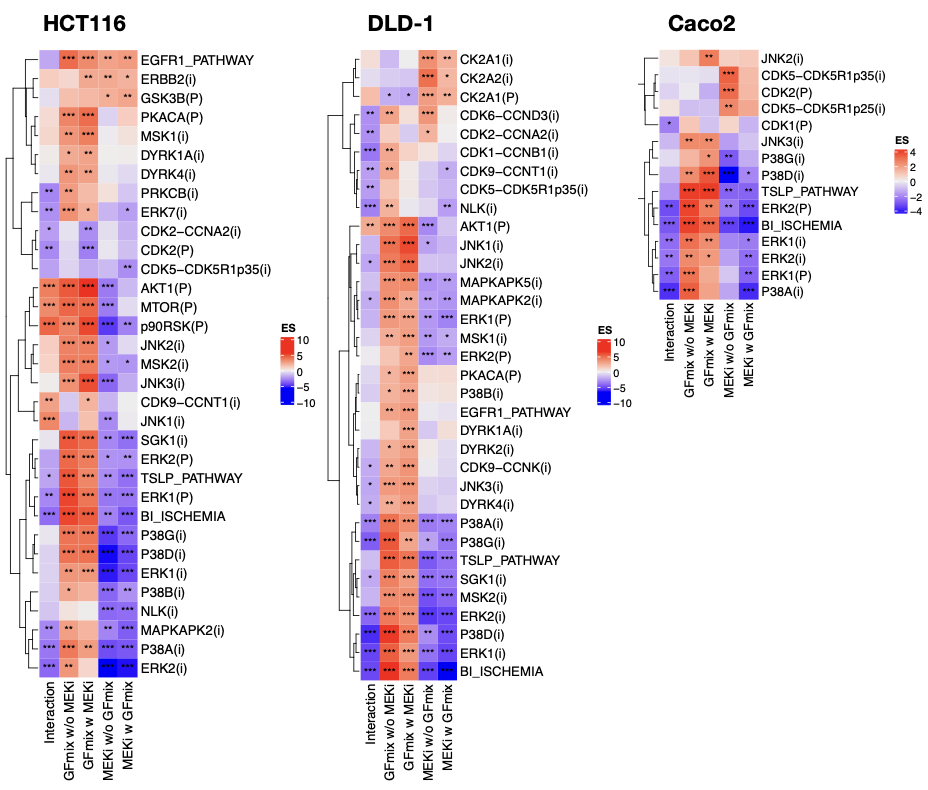
**Supplementary Figure 13. Complete results from PTM-SEA.** Signatures derived from PhosphoSitePlus (PSP) and iKiP-DB denoted by (P) and (i), respectively. The input for PTM-SEA consists of fold-change signed p-values from a moderated t-test, specifically filtered for phospho-peptides with an moderated F-test p-value <0.1, indicating significant regulation in at least one of the tests. ES = enrichment score as calculated by PTM-SEA. Significance is denoted by asterisks, with * = 0.1, ** 0.05, *** = 0.01.


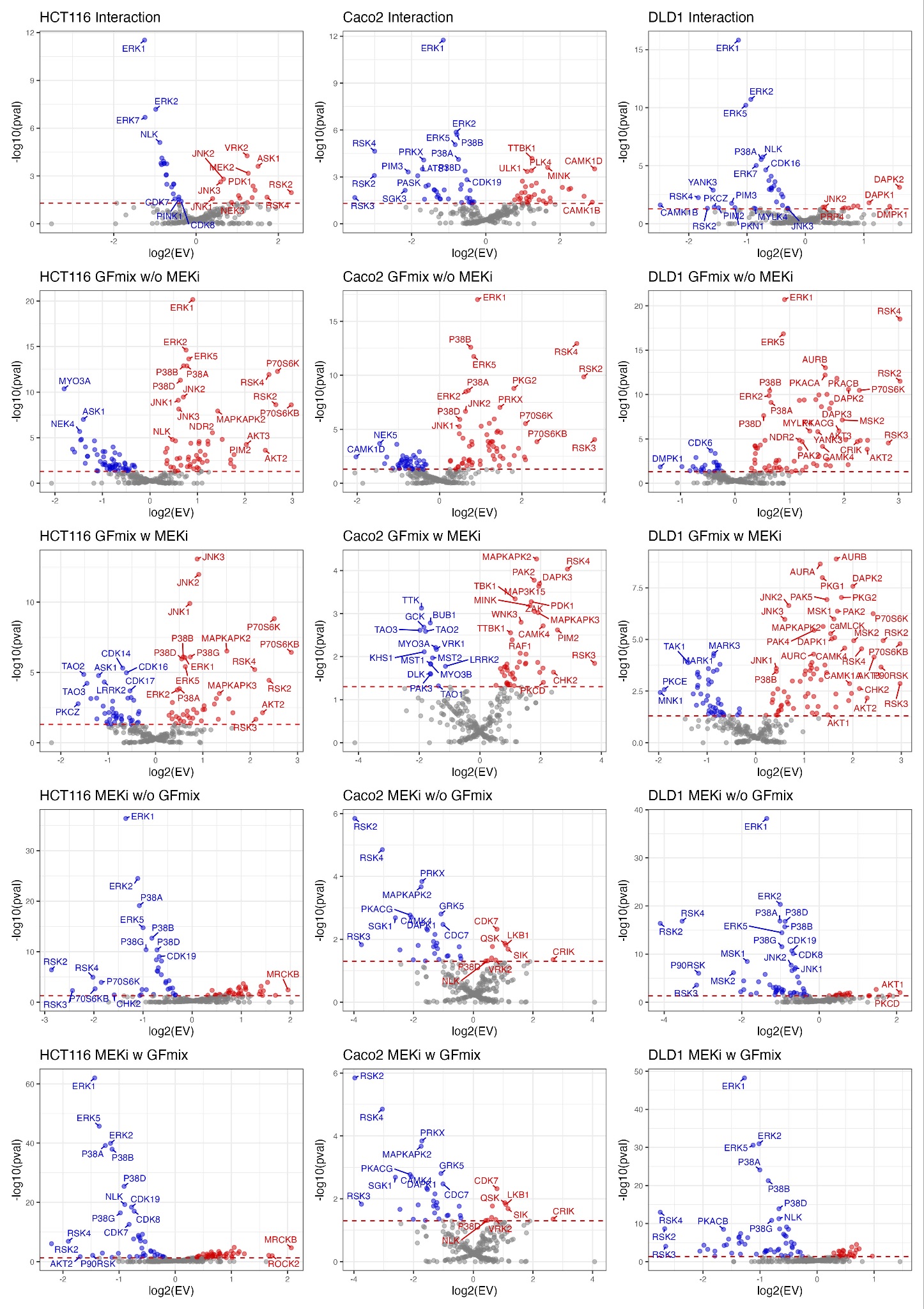


**Supplementary Figure 14. Kinase Library S/T (Serine/Threonine) Kinase Motif Enrichment Analysis.** For each comparison, input phosphopeptides were selected with a fold change (FC) > 0.1 and a p-value < 0.05, as foreground phosphosites. The output is the enrichment value (EV).


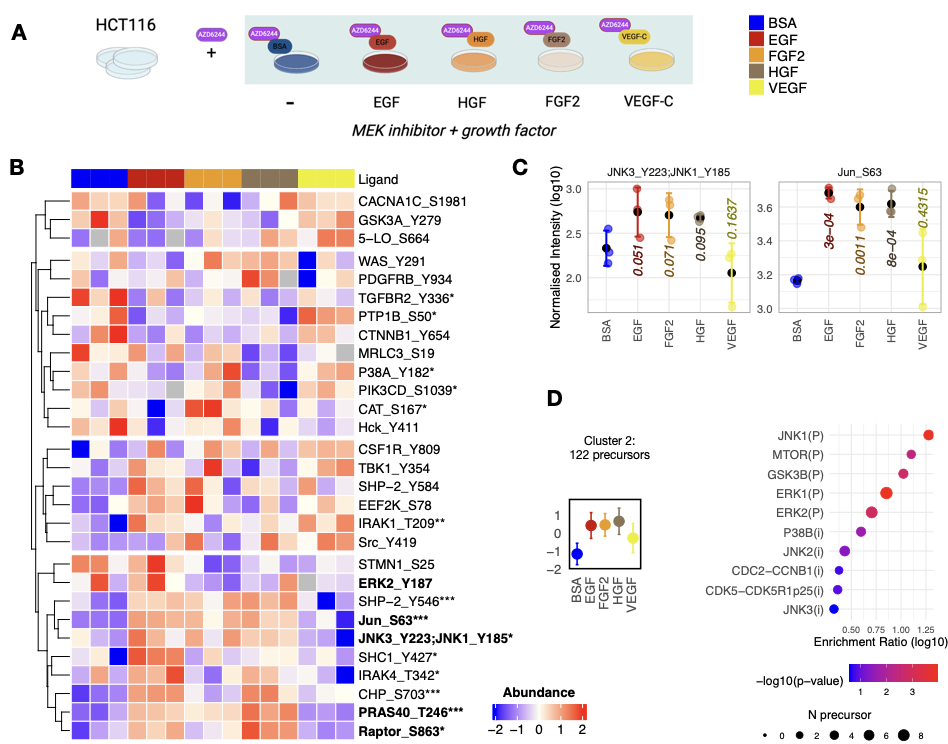


**Supplementary Figure 15: Phosphoproteomic Analysis of HCT116 Cells with MEKi and Individual Growth Factor Treatment.** A. Schematic of the experimental workflow with consistent color coding across the figure. B. Targeted analysis results: Heatmap presenting phosphorylation site regulation under various conditions, filtered by F-test significance (p < 0.2). The color scale reflects z-normalized abundance, with asterisks denoting F test p-values: *** for p < 0.01, ** for p < 0.05, and * for p < 0.1. C. Normalized intensity as derived from targeted analysis plots for select phosphorylation sites, loess-normalized and log10-transformed. Standard deviations are depicted as error bars. p-values as derived from limma moderated t-test (BSA~GF) are depicted. D. Hierarchical clustering of phosphopeptides of label-free data based on mean intensity profiles per treatment, error bars indicating standard deviation. Enrichment analysis of PhosphoSitePlus and iKiP-DB kinase signature for cluster 2.


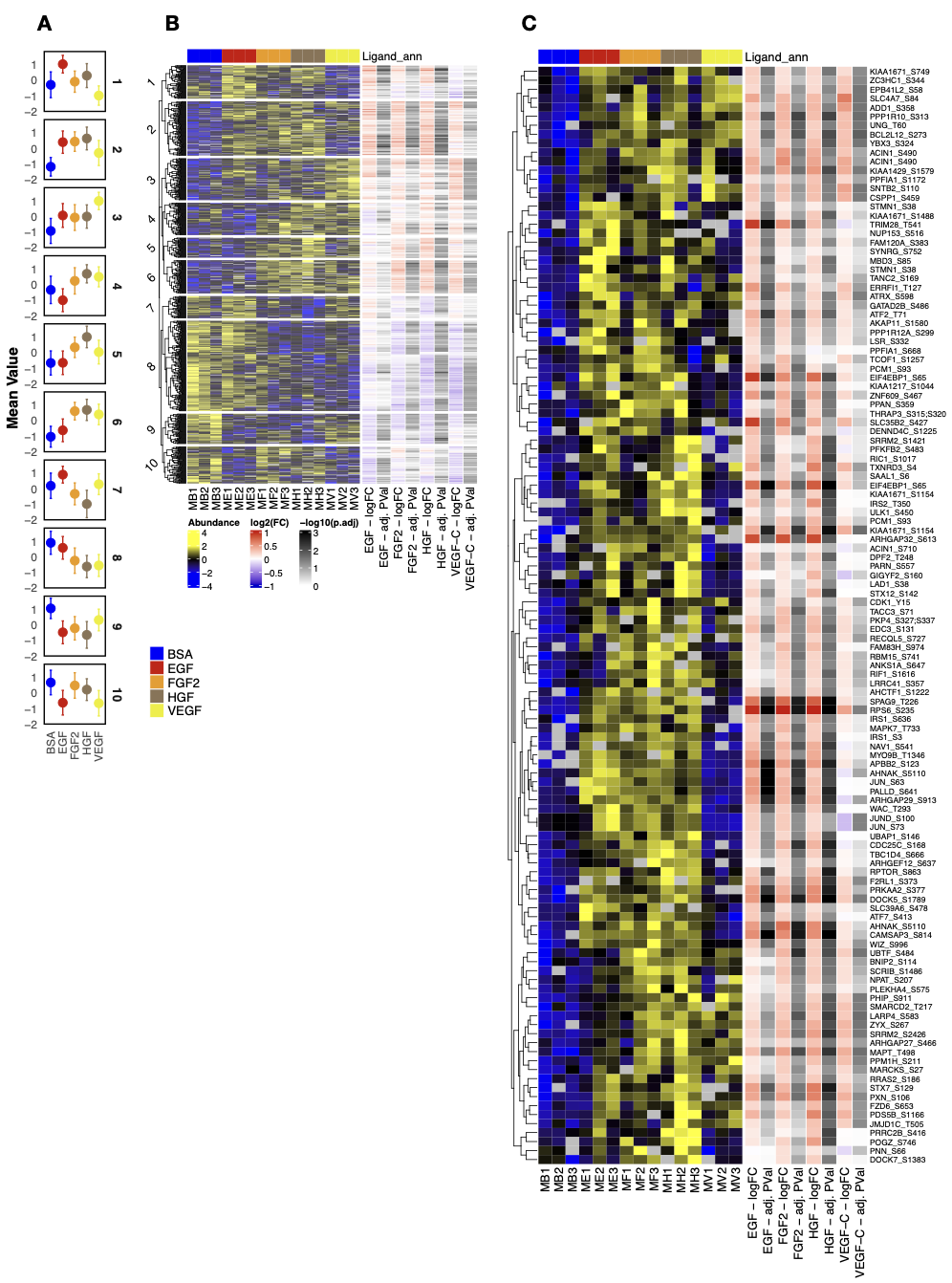
**Supplementary Figure 16. Hierarchical Clustering and Differential Abundance Analysis of phosphopeptides in label-free data HCT116 with individual growth factors.** A. Mean intensity profiles per treatment condition across identified clusters. Color coding consistent with heatmap in panel B/C. Error bars represent standard deviation. B. Heatmap of significantly regulated phosphosites (moderated F test p-value < 0.1) from HCT116 label-free data. Row-wise z-score normalization applied to precursor intensities. Column to the right depict log2 fold change and BH adjusted p-values (-log10) derived from the limma moderated t-test. C. Detailed view of cluster 1. The column to the right shows log2 fold change and -log10(BH adjusted p-value) associated with the limma t-tests.
