# Supplementary material for: Spike-in enhanced phosphoproteomics uncovers synergistic signaling responses to MEK inhibition in colon cancer cells
Source: Nat Commun. 2025 May 27;16:4884. doi: 10.1038/s41467-025-59404-y (PMC12106795; doi:10.1038/s41467-025-59404-y)
Supplement: Supplementary file 2 — Reporting Summary [file 41467_2025_59404_MOESM2_ESM.pdf]

nature portfolio

Corresponding author(s): Matthias Selbach, Nils Bluthgen

Last updated by author(s): Jan 8, 2025

Reporting Summary

Nature Portfolio wishes to improve the reproducibility of the work that we publish. This form provides structure for consistency and transparency in reporting. For further information on Nature Portfolio policies, see our Editorial Policies and the Editorial Policy Checklist.

Statistics

For all statistical analyses, confirm that the following items are present in the figure legend, table legend, main text, or Methods section.

n/a	Confirmed	
☐	☒	The exact sample size (*n*) for each experimental group/condition, given as a discrete number and unit of measurement
☐	☒	A statement on whether measurements were taken from distinct samples or whether the same sample was measured repeatedly
☐	☒	The statistical test(s) used AND whether they are one- or two-sided<br>*Only common tests should be described solely by name; describe more complex techniques in the Methods section.*
☒	☐	A description of all covariates tested
☐	☒	A description of any assumptions or corrections, such as tests of normality and adjustment for multiple comparisons
☒	☐	A full description of the statistical parameters including central tendency (e.g. means) or other basic estimates (e.g. regression coefficient) AND variation (e.g. standard deviation) or associated estimates of uncertainty (e.g. confidence intervals)
☐	☒	For null hypothesis testing, the test statistic (e.g. *F*, *t*, *r*) with confidence intervals, effect sizes, degrees of freedom and *P* value noted<br>*Give P values as exact values whenever suitable.*
☒	☐	For Bayesian analysis, information on the choice of priors and Markov chain Monte Carlo settings
☒	☐	For hierarchical and complex designs, identification of the appropriate level for tests and full reporting of outcomes
☒	☐	Estimates of effect sizes (e.g. Cohen's *d*, Pearson's *r*), indicating how they were calculated

Our web collection on statistics for biologists contains articles on many of the points above.

Software and code

Policy information about availability of computer code

Data collection	No specific code was used for data collection
Data analysis	Data analysis was performed with software tools (MaxQuant v2.4.0.0 and DIA-NN v1.8.2 beta 11). MaxQuant and DIA-NN output were processed in R (v4.3.0). Normalisation and differential abundance analysis were performed using the limma package (v3.56.1). Raw data and spectra were manually inspected using the Bruker Data Analysis tool.<br>The R scripts necessary to perform SPIED-DIA analysis, as well as the scripts to perform the analyses relevant to the main figures in the manuscript can be found at https://github.com/Mirjamva/SPIED-DIA. The repository was linked to Zenodo (https://doi.org/10.5281/zenodo.15045498)

For manuscripts utilizing custom algorithms or software that are central to the research but not yet described in published literature, software must be made available to editors and reviewers. We strongly encourage code deposition in a community repository (e.g. GitHub). See the Nature Portfolio guidelines for submitting code & software for further information.

Data

Policy information about availability of data

All manuscripts must include a data availability statement. This statement should provide the following information, where applicable:

- Accession codes, unique identifiers, or web links for publicly available datasets
- A description of any restrictions on data availability
- For clinical datasets or third party data, please ensure that the statement adheres to our policy

Mass spectrometry raw files as well as MaxQuant and DIA-NN output files, spectral libraries and associated files have been deposited to the ProteomeXchange Consortium via the PRIDE partner repository. The accession ID is PXD050961 [https://www.ebi.ac.uk/pride/archive/projects/PXD050961]. The processed data such as normalized intensities and results of the differential expression analysis as derived from the proteomics experiments are available as Supplementary Data files. The raw data of the Bio-Plex and live-cell imaging experiments are provided as Supplementary Data files.

Research involving human participants, their data, or biological material

Policy information about studies with human participants or human data. See also policy information about sex, gender (identity/presentation), and sexual orientation and race, ethnicity and racism.

Reporting on sex and gender	n/a
Reporting on race, ethnicity, or other socially relevant groupings	n/a
Population characteristics	n/a
Recruitment	n/a
Ethics oversight	n/a

Note that full information on the approval of the study protocol must also be provided in the manuscript.

Field-specific reporting

Please select the one below that is the best fit for your research. If you are not sure, read the appropriate sections before making your selection.

☒ Life sciences ☐ Behavioural & social sciences ☐ Ecological, evolutionary & environmental sciences

For a reference copy of the document with all sections, see nature.com/documents/nr-reporting-summary-flat.pdf

Life sciences study design

All studies must disclose on these points even when the disclosure is negative.

Sample size	The sample size was set to 4 biological replicates for the Bio-Plex experiment and 3 biological replicates for all other experiments.
Data exclusions	During the Bio-Plex data-processing, obvious outliers among replicates (absolute z-score >=3 for all three phospho-sites measurments) were removed. For LC-MS measurements, one sample was lost during sample preparation (belonging to DLD-1 GFmix group) and one sample was excluded due to disappointing MS performance (Caco2, belonging to MEKi condition).<br>During data-processing of the cell-growth imaging experiment, raw growth curves were visually scanned for outliers.
Replication	4 biological replicates were collected per experimental group for the Bio-Plex experiements. For all LC-MS measurements we aimed to collect 3 biological replicates. For the cell-growth curve experiments 3 biological replicates were collected. 4 images were captured per well. Technological replicates were included for the control conditions
Randomization	The order of within experiment LC-MS measurements was randomized to ensure any variance due to (small) changes in chromatography or machine performance would not be reflected in the experimental groups.
Blinding	Not relevant: data acquisition and analysis were performed in an automated way.

Reporting for specific materials, systems and methods

We require information from authors about some types of materials, experimental systems and methods used in many studies. Here, indicate whether each material, system or method listed is relevant to your study. If you are not sure if a list item applies to your research, read the appropriate section before selecting a response.

Materials & experimental systems

n/a	Involved in the study
☐	☒ Antibodies
☐	☒ Eukaryotic cell lines
☒	☐ Palaeontology and archaeology
☒	☐ Animals and other organisms
☒	☐ Clinical data
☒	☐ Dual use research of concern
☒	☐ Plants

Methods

n/a	Involved in the study
☒	☐ ChIP-seq
☒	☐ Flow cytometry
☒	☐ MRI-based neuroimaging

Antibodies

Antibodies used	We used bead-bases ELISAs, called Bio-plex pro Phosphoprotein magnetic 3-plex Assay (BioRad), containing antibodies against Akt (Ser473), Erk2 (Thr185/Tyr187), MEK1 (Ser217/Ser221). Kit number LQ0000000001ZG
Validation	LISAs were validated by BioRad, as well as in [https://doi.org/10.1371/journal.pcbi.1009515], where we perturbed cells with small molecule inhibitors targeting MEK and PI3K which validated the response of the ELISAs.

Eukaryotic cell lines

Policy information about cell lines and Sex and Gender in Research

Cell line source(s)	Human colorectal cell-lines used in the Bio-Plex screen (Colo205, Colo678, DLD-1, GEO, HCT116, HT29, LIM1215, RKO, SW403, SW480 and Caco-2) were provided by AG Sers Molekulare Tumorpathologie (Charité-Universitätsmedizin). All other experiments were performed using HCT116, Caco2 and DLD-1 directly obtained from ATCC (Manassas, Virginia, USA).
Authentication	The human colorectal cell-lines used in the Bio-Plex sceen and cell lines directly obtained from ATCC were authenticated via PCR-single-locus technology
Mycoplasma contamination	All cell lines were tested negative for mycoplasma contamination.
Commonly misidentified lines (See ICLAC register)	n/a

Plants

Seed stocks	*Report on the source of all seed stocks or other plant material used. If applicable, state the seed stock centre and catalogue number. If plant specimens were collected from the field, describe the collection location, date and sampling procedures.*
Novel plant genotypes	*Describe the methods by which all novel plant genotypes were produced. This includes those generated by transgenic approaches, gene editing, chemical/radiation-based mutagenesis and hybridization. For transgenic lines, describe the transformation method, the number of independent lines analyzed and the generation upon which experiments were performed. For gene-edited lines, describe the editor used, the endogenous sequence targeted for editing, the targeting guide RNA sequence (if applicable) and how the editor was applied.*
Authentication	*Describe any authentication procedures for each seed stock used or novel genotype generated. Describe any experiments used to assess the effect of a mutation and, where applicable, how potential secondary effects (e.g. second site T-DNA insertions, mosiacism, off-target gene editing) were examined.*
