# Supplementary material for: Spike-in enhanced phosphoproteomics uncovers synergistic signaling responses to MEK inhibition in colon cancer cells
Source: Nat Commun. 2025 May 27;16:4884. doi: 10.1038/s41467-025-59404-y (PMC12106795; doi:10.1038/s41467-025-59404-y)
Supplement: Supplementary file 3 — Description of Additional Supplementary Files [file 41467_2025_59404_MOESM3_ESM.docx]

**File Name: SupplementaryData1_SelectedPhosphopeptides.csv**

Selected phosphopeptides for heavy spike-in. (Underlying Figure 1F)

**File Name: SupplementaryData2_IdentfiedSILpeptides.csv**

Heavy-labeled phosphopeptide identifications used for analysis underlying Figures 3–4.

**Filename: SupplementaryData3_BioPlexRawData.csv**

This contains the raw data as derived from the BioPlex assay underlying Figure 2 and Supplementary Figure 6. Source data for Figure 2BD is provided as well.

**Filename: SupplementaryData4_allCellLines_SPIEDDIA_NormInt.csv**

Normalised intensities as produced by the SPIED-DIA workflow for all cell lines (Figures 3, 4). This was generated by processing DIA-NN-derived phosphoproteomic data for targeted phosphopeptide analysis of the experiment as described in figure 3. Specifically, phosphopeptide precursor intensities were extracted from the DIA-NN report (report.tsv), filtered as described in materials and methods.

Intensity values were log-transformed, normalization was subsequently performed separately for each cell line (HCT116, DLD-1, and Caco2), each with three biological replicates per treatment condition (exceptions for DLD-1 GFmix/DMSO and and Caco2 BSA/MEKi, n=2). The resulting table shows log-transformed, cyclic-loess-adjusted intensity values for each peptide precursor across the experimental conditions.

The raw data for this dataset are provided on PRIDE (PanelGFmix_Rawfiles.zip), as is the DIA-NN output file (PanelFGmix_outputDIANN_SPIED.zip) as well as the associated spectral library files and the analysis script is posted on GitHub (GitHub_SPIED-DIA/Manuscipt_scripts/Biological/CellLinePanel_Targeted/CellLinePanel_TargetedAnalysis.R)

**Filename: SupplementaryData[5 | 6 | 7]_[HCT116 | DLD-1 | Caco2]_SPIEDDIA_limma_results.csv**

These tables, one per cell line contain differential abundance results derived from the limma differential expression analysis applied to the normalized SPIED-DIA data. Rows correspond to peptide precursors, with columns giving adjusted p-values, fold changes, and F‑test statistics for various contrasts (LigandMix vs. BSA, MEKi vs. DMSO, and synergistic terms).

Generated by CellLinePanel_TargetedAnalysis.R script (available on GitHub_SPIED-DIA/Manuscipt_scripts/Biological/CellLinePanel_Targeted/CellLinePanel_TargetedAnalysis.R). Heatmap data sets (SourceData_Figure4A.csv, SourceData_Figure4B.csv, SourceData_Figure4C.csv) exhibit scaled (z-score) abundance changes for notable phosphosites under F-test threshold 0.1. Figures 4ABCE and Supplementary Figure 9 are generated from this data.

**Filename: SupplementaryData8_allCellLines_Global_NormInt.csv**

Normalised intensities as produced by the global analysis workflow (Figures 3, 5). This table is generated by filtering, log10 transformation, and cyclic Loess normalization of the DIA-NN output file. Each row represents a single precursor, while each column provides the normalized log10 intensity for a specific sample or run.

The raw data for this dataset are provided on PRIDE (PanelGFmix_Rawfiles.zip), as is the DIA-NN output file (PanelGFmix_outputDIANN_LF.zip) as well as the associated spectral library files and the analysis script is posted on GitHub (available on GitHub under GitHub_SPIED-DIA/Manuscipt_scripts/Biological/CellLinePanel_Global/CellLinePanel_Global_Norm.R). This script generates Figure 3CD (source data provided).

It is the primary input for DiffExpression.R script that performs the differential expression analysis and visualisation of associated manuscript figures.

**Filename: SupplementaryData[9 | 10 | 11]_[HCT116 | DLD-1 | Caco2]_Global_limma_results.csv**

These tables, one per cell line contain differential abundance findings derived from the limma differential expression analysis applied to the normalized global data (NormInt_Global_allCellLines.csv). Rows correspond to peptide precursors, with columns giving adjusted p-values, fold changes, and F‑test statistics for various contrasts (LigandMix vs. BSA, MEKi vs. DMSO, and synergistic terms). The associated script (GitHub_SPIED-DIA/Manuscipt_scripts/Biological/CellLinePanel_Global/DiffExpression.R) performs the differential expression analysis and produces Figure 5A (dependent on script GitHub_SPIED-DIA/Manuscipt_scripts/Biological/CellLinePanel_Global/DiffExpression.R) and Supplementary Figures 10, 11, 12. The results are further analysed to produce Figure 5B and 5C.

**Filename: SupplementaryData12_HpHlibrary_annotation.csv**

Annotation file HpH library used in the analysis global data. Necessary for script analysing the global data.

**Filename: SupplementaryData13_KinLibIn.csv**

This CSV file contains moderated t‑test output (fold changes and p‑values) for each phosphosite comparison as generated by the limma differential expression analysis of the global data. Code to format these tables as required by the S/T kinase library tool (https://kinase-library.phosphosite.org/) is provided in the associated GitHub repository under “GitHub_SPIED-DIA/Manuscipt_scripts/Biological/CellLinePanel_Global/Cantleykinase_analysis.R”. These results underlie Figure 5C and Supplementary Figure 14.

**Filename: SupplementaryData14_KinLibOut.csv**

Collects final outcomes from the S/T kinase library tool across all group × cell line comparisons. It indicates predicted kinase motifs, their statistics, and overall significance. The script for generating volcano plots in Figure 5C and Supplementary Figure 14 is found in GitHub_SPIED-DIA/Manuscipt_scripts/Biological/CellLinePanel_Global/Cantleykinase_analysis.R, with matching source data provided as well.

**Filename: SupplementaryData[15 | 16]_LiveCellImaging_MEKi[PI3ki | JNKi]_RawData.csv**

Description: Raw data (as derived from Incucyte software) underlying Figure 6. Source data for these figures is provided as well.
